# Growth and mineralization of fetal mouse long bones under microgravity and daily 1 g gravity exposure

**DOI:** 10.1038/s41526-024-00421-4

**Published:** 2024-07-27

**Authors:** Jack J. W. A. van Loon, Olga P. Berezovska, Theodorus J. M. Bervoets, Dina Montufar-Solis, Cor M. Semeins, Behrouz Zandieh-Doulabi, P. Natalia V. Rodionova, Jackie Duke, J. Paul Veldhuijzen

**Affiliations:** 1 Department of Oral Biology, Section Oral Cell Biology, ACTA–Vrije Universiteit, Amsterdam, The Netherlands; 2 Department of Radiobiology and Radioecology, Institute for Nuclear Research of National Academy of Sciences of Ukraine, Kiev, Ukraine; 3 Department of Integrative Biology and Pharmacology, McGovern Medical School, University of Texas Health Science Center, Houston, TX USA; 4 Schmalhausen Institute for Zoology, National Academy of Sciences Ukraine, Kiev, Ukraine; 5 Department of Orthodontics & Dentofacial Orthopedics, University of Texas Health Science Center, Houston, TX USA

**Keywords:** Physiology, Biophysics

## Abstract

In a previous Space Shuttle/Spacelab experiment (STS-42), we observed direct responses of isolated fetal mouse long bones to near weightlessness. This paper aimed to verify those results and study the effects of daily 1×*g* exposure during microgravity on the growth and mineralization of these bones. Two experiments were conducted: one on an American Space Shuttle mission (IML-2 on STS-65) and another on a Russian Bio-Cosmos flight (Bion-10 on Cosmos-2229). Despite differences in hardware, both used 17-day-old fetal mouse metatarsals cultured for 4 days. Results showed reduced proteoglycan content under microgravity compared to 1×*g* conditions, with no main differences in other cellular structures. While the overall metatarsal length was unaffected, the length increase of the mineralized diaphysis was significantly reduced under microgravity. Daily 1×*g* exposure for at least 6 h abolished the microgravity-induced reduction in cartilage mineralization, indicating the need for long-duration exposure to 1×*g* as an in-flight countermeasure using artificial gravity.

## Introduction

It is well documented that under microgravity conditions, bone mass decreases, sometimes dramatically, in humans and experimental animals (for reviews, see refs. ^1–6^). During the period the here presented studies were performed (1993–1994) only a few isolated skeletal tissues and cell studies^7–12^ had been flown on the American Space Shuttle or Russian Biosatellites. The results of these experiments suggested that changes in bone and cartilage metabolism of humans and rats might be, at least partly, explained by the direct effects of microgravity on bone and cartilage cells. Although it is currently improving, a major drawback of all microgravity experiments continues to be the scarcity of flight opportunities for verification of results. The current paper reports a unique opportunity: two microgravity experiments were performed in which the same experimental model and, in one case, the same culture conditions were used, allowing comparison with a previous study^11^.

Especially for bone, there are a number of reports that address the possibility that a memory mechanism for mechanical loading events may exist in skeletal tissues^13–17^. There are also reports that a relatively low number of daily repeated loading events is sufficient to fully reduce immobilization-induced bone loss^18,19^. A more recent study using bone marrow cells showed that a structural mechanical memory effect in these cells might be preserved through the excretion of extracellular vesicles^20^. This mechanical memory effect could contribute to preventing reduced bone matrix mineralization induced by long-duration microgravity both in vivo and in vitro. One concept is that such a load could be provided by short daily exposure to gravity via short-arm centrifugation^21,22^.

This hypothesis was tested in an in vitro system where one of the microgravity experiments, in which the isolated fetal long bones were exposed to various periods of daily 1×*g*, was designed to study the presence of a tissue memory in relation to matrix mineralization. Results of this experiment can shed light on possible effects of the applications of an in-flight 1×*g* centrifuge as (multisystem) countermeasure device. Also, the validity of an in-flight control group is of crucial importance in evaluating the data of microgravity groups.

## Results

### IML-2: STS-65

**Fig. 1:**
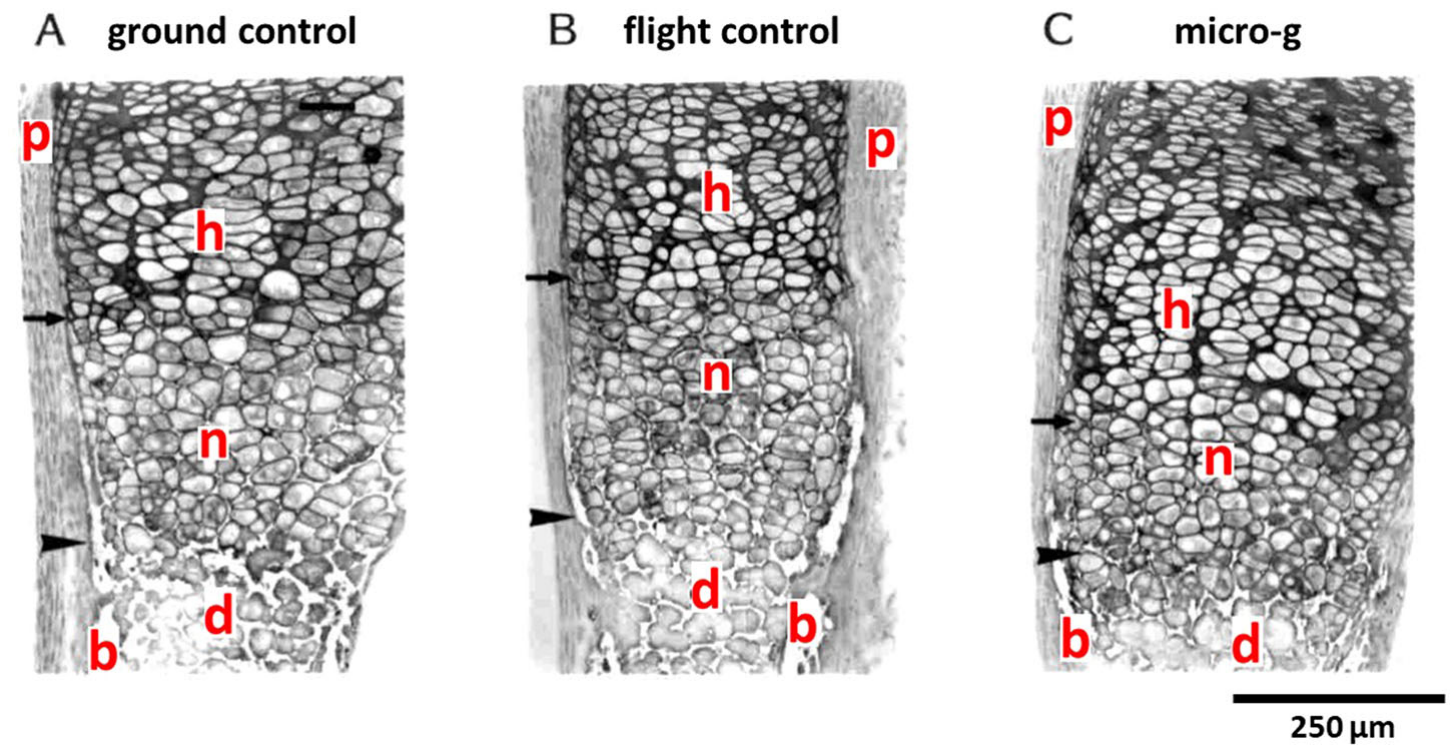
Photomicrographs of histological sections of fetal mouse metatarsal long bones (IML-2 mission). **A** Ground control (4 days culture), **B** flight control (4 days culture on in-flight 1×*g* centrifuge) and **C** microgravity (4 days culture). d = mineralized diaphysis at *T* = 0; n = newly mineralized diaphysis. The newly mineralized zone can be identified as adjacent to the hypertrophic cartilage zone (h). The newly mineralized zone can be distinguished from the diaphysis by still-intact chondrocytes with a mineralized matrix around them. b developing bone collar, p perichondrium, h hypertrophic cartilage. Arrowhead = mineralization front at *T* = 0 day; Arrow = mineralization front at day 4. Bar is 50 µm (for all photomicrographs).

**Fig. 2:**
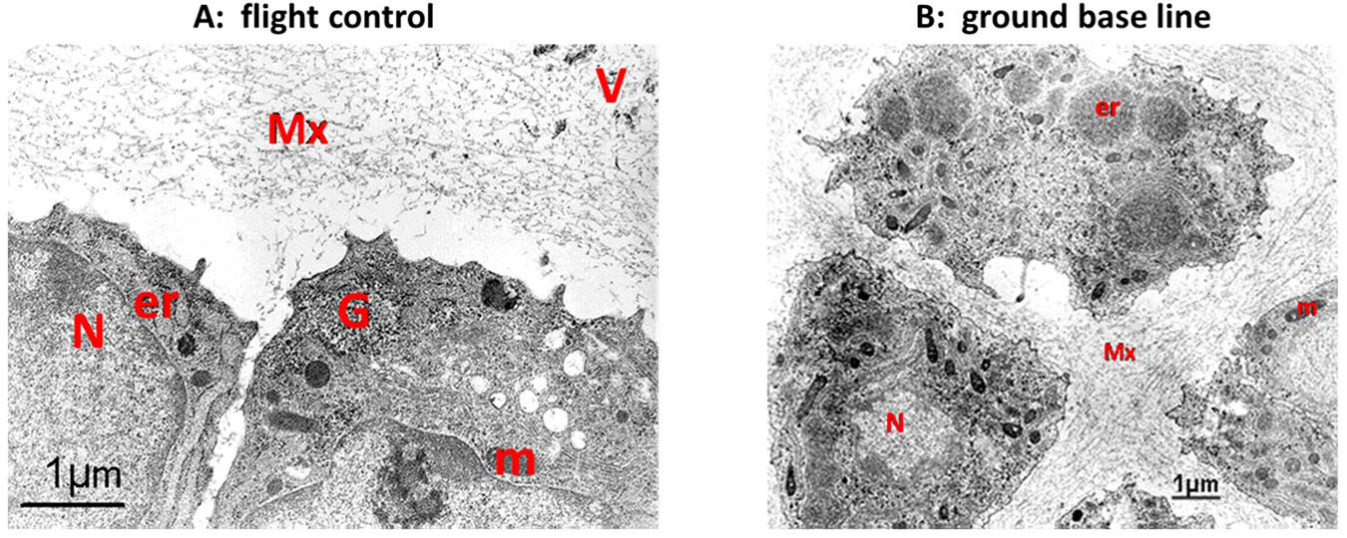
An electron microscopy photomicrograph of chondrocytes exposed to microgravity. **A** Chondrocytes in reserve zone of an in-flight control (1 × *g* centrifuge) metatarsal long bone (IML-2 mission). **B** Chondrocytes in the reserve zone of a metatarsal long bone from the baseline group (prior to culture) (IML-2 mission). Notice the organization of the extracellular matrix which is much more structured in the pre-cultured sample. N nucleus, m mitochondria, er endoplasmic reticulum, Mx extracellular matrix, V matrix vesicles, G glycogen.

Light microscopy evaluation of the metatarsals showed healthy chondrocytes surrounded by a normal-looking matrix. No pycnotic cells were observed. No differences in the morphology of the tissues were seen between the ground control group (Fig. 1A), the flight control group (Fig. 1B), and the microgravity group (Fig. 1C). In a very small number of long bone resorption areas were seen in the center of the mineralized diaphysis. Using the TRAcP-staining, osteoclasts were detected in the diaphysis of only a few long bones. Ultrastructural analyses revealed that cells in all groups showed the high nuclear/cytoplasmatic ratio typical of chondrocytes, contained large amounts of glycogen, and numerous mitochondria associated with the RER. Numerous vesicles were observed throughout the extracellular matrix of all tissues (Fig. 2A, B). Cell division was observed in both horizontal and vertical axes throughout the developing metatarsal, with less organized fibrils in the more actively proliferating areas. Collagen fibrils in the reserve zone showed a wide range of sizes for all groups, with no evident differences in size or orientation. In the proliferative and hypertrophy zones, the fibrils appeared more oriented in all groups, but there were no other obvious distinctions between the groups. Proteoglycan granules differed slightly between the groups exposed to 1×*g*, either baseline (which appeared to have the most), flight control, and ground control groups, but in general, all of these groups had apparently more granules than the microgravity group.

**Fig. 3:**
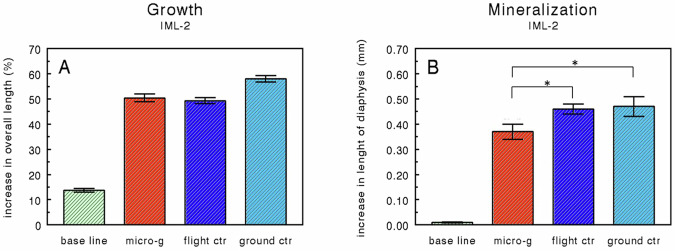
Metatarsal growth and mineralization in near weightlessness. Percentage increases in overall length (**A**) and absolute increases (mm) in the length of the mineralized diaphysis (**B**) of fetal mouse long bones (IML-2 mission) compared L-2 days and post-flight (see also Table 1). Values are presented as means ± SEM. **A** *n* = 16; micro-g: *n* = 15; **B** *n* = 16; micro-g: *n* = 15. Significant differences between groups (*p* < 0.05) are indicated by an asterisk (*). The baseline group is significantly different from all other groups.

All metatarsals increased considerably in length during the 4-day experimental period at 37 °C (Fig. 3A). The increase in the baseline group, as measured after an overnight pre-culture period at 37 °C followed by a 24-h period at room temperature, was about 15%. After an additional 4 days of culture, all metatarsals increased in overall length by about 50%. No difference was found between the microgravity and the flight control group. The overall length increase of the flight control group (1×*g* in flight) did not differ significantly from the 1×*g* ground control group.

**Fig. 4:**
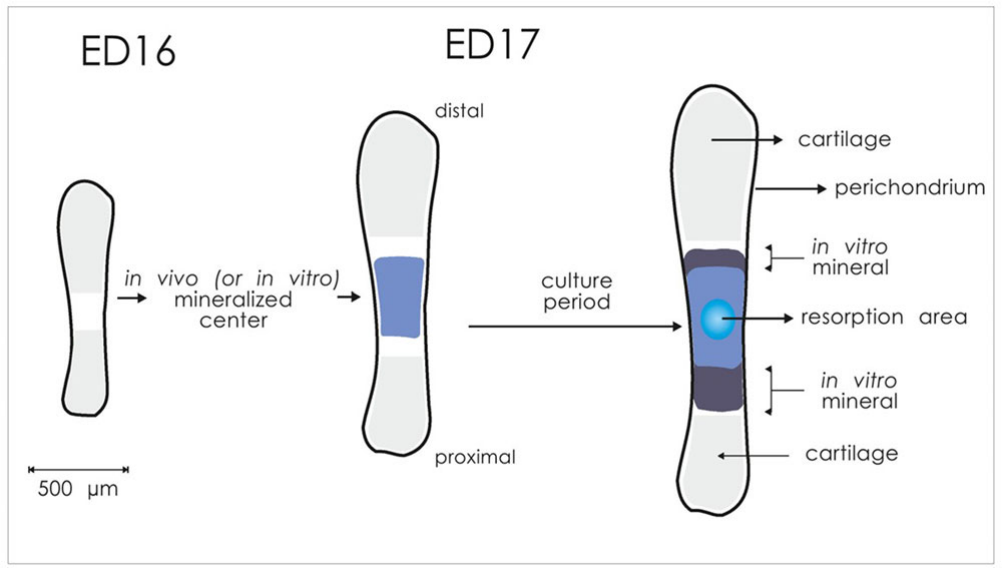
Graphical display of an Embryonic Day 16 (ED16) and ED17 fetal mouse metatarsal long bones. For the IML-2 and Bion-10 experiments, we used 17 days old bones at the time of dissection. The ED17 rudiments are about 1.5–2 mm long at dissection. Before culture, the central mineralized zone (the diaphysis) has developed in vivo. It is flanked by zones of cartilage (the epiphysis). The whole matrix is surrounded by a thin layer of perichondrium. During culture, there is an increase in total length as well as an increase in the length of the diaphysis (in vitro mineral). In some bones, a more translucent resorption area can be seen after some time.

At the beginning of the experiment, all metatarsals were already mineralized in vivo (see Fig. 4). During the overnight culture (without Na-ß-glycerophosphate) followed by the 24 h period at room temperature (Na-ß-glycerophosphate included), mineralization only marginally progressed (<0.1 mm) as is seen in Fig. 3B (baseline group). In the microgravity group, mineralization was significantly retarded compared to the flight control group (Fig. 3B). No difference in the length of the mineralized zone was found between the flight control and the ground control. When the total length of the zone of hypertrophic cartilage was calculated, no significant differences were found between the microgravity and the flight control group (data not shown).

**Fig. 5:**
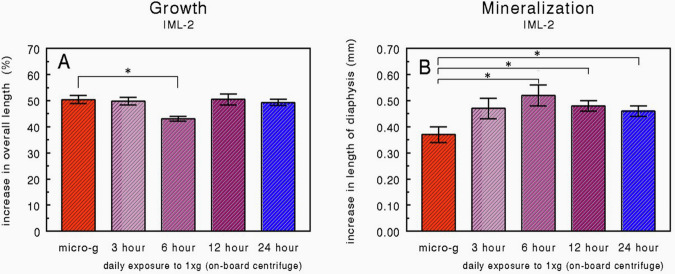
Metatarsal growth and mineralization in near weightlessness and various periods of 1 × *g* exposure. Difference between measurement of L-2 days and post-flight in percentage increases in overall length (**A**) and absolute increases (mm) in the length of the mineralized diaphysis (**B**) of fetal mouse long bones cultured for 4 days under microgravity, and under microgravity alternated with daily exposure to 1 × *g* (IML-2 mission). The 24-h centrifuge group is the flight control. Values are presented as means ± SEM. **A** *n* = 16; micro-*g* and 6 h: *n* = 15; **B** *n* = 16; micro-*g* and 6 h: *n* = 15. Significant differences between the micro-*g* group and the centrifuge groups (*p* < 0.05) are indicated by an asterisk (*).

Placing the long bones for varying daily periods on the on-board 1×*g* centrifuge did not affect the overall increase in length seen in the flight experiment (Fig. 5A). Only in the group exposed daily for 6 h to 1×*g*, was a slight but significant retarded growth observed, compared to the microgravity group and the flight control group.

Daily interruption of microgravity conditions by exposure to 1×*g* in-flight centrifugation for 6 or *12 h completely restored the mineralization to the level of the flight control (24 h on the 1×*g* centrifuge for 4 days) (Fig. 5B). These three groups all showed significantly greater mineralization than the microgravity group. Exposure for only 3 h to 1×*g* conditions each day resulted in a slight, but not significant increase in mineralization compared to the microgravity group. However, first-order regression analysis of all data, with the 1×*g* exposure time as a variable, showed a significant (*p* < 0.05) increase in the mineralization. So the increase in mineralization correlates directly with the increasing hours of exposure to 1×*g* centrifugation.

### Bion-10: Cosmos-2229

**Fig. 6:**
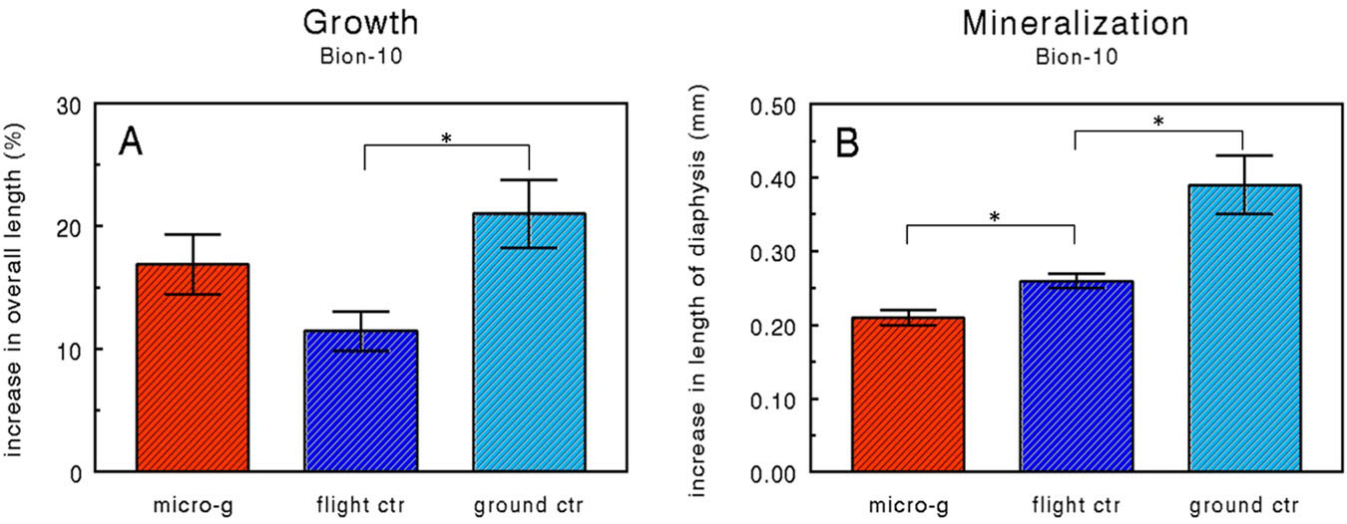
Metatarsal growth and mineralization in near weightlessness during Cosmos 2229 mission. Percentage increase in overall length (**A**) and absolute increase (mm) in the length of the mineralized diaphysis (**B**) of fetal mouse long bones cultured for 4 days in Biobox (Bion-10 mission) under microgravity or on the on-board 1 × *g* centrifuge (flight ctr.) and in the ground model of Biobox under Earth’s 1 × *g* gravity (ground ctr.). Values are presented as means ± SEM. **A** and **B**: micro-*g*: *n* = 6; flight ctr.: *n* = 12; ground ctr.: *n* = 9. Significant differences between groups (*p* < 0.05) are indicated by an asterisk (*).

About two-third of the flight samples did not increase in length, and/or histological evaluation showed necrotic cells in many of such rudiments. Only those rudiments that did show growth and had no or only limited signs of necrotic cells were included in the final analyses. The metatarsals included showed a moderate percentage increase in length (growth), ranging from 11.5% in the flight control to 21% in the ground control. Length increase in the microgravity group (17%) did not differ significantly from the flight control (Fig. 6A). However, the flight control had a significantly reduced percentage length increase compared to the ground control. Also, the mineralization of the diaphysis, measured as the absolute increase in the length of the mineralized zone, was very moderate (Fig. 6B). Measurements showed that under microgravity the mineralization was significantly less as compared to the flight control group (Fig. 6B). There was also a significant difference between the mineralization in the flight control and the ground control.

## Discussion

Although the biological material of these two microgravity experiments was the same, the hardware and the flight conditions were different. For the IML-2 experiment, we could use the proven hardware and experiment scenario of the successful IML-1 mission^11^. The culture in polyethylene gas-permeable culture bags mounted in Biorack Type-I containers, which are flushed twice during the mission with a gas mixture of 5% CO_2_ in the air, is very reliable and does hardly differ from the culture in standard 24-well culture plates^23^.

The plunger box principle of the Bion-10 hardware that allowed for predetermined, automatic liquid changes was developed originally for microgravity experiments with *Xenopus laevis* eggs^24^. Although adapted for tissue culture, the culture of the metatarsals in this hardware never equaled the results in standard multiwell cultures (unpublished results), indicating that the culture conditions in the Bion-10 hardware were indeed sub-optimal. Sub-optimal growth conditions, especially in the in-flight samples, were also reported by Klement and co-workers^25^. However, this might have been caused by limited culture conditions in flight due to, e.g. reduced gas exchange.

A second drawback in the Bion-10 experiment was the long period between the handover of samples to ESA officials for integration into Biobox and the actual start of the experiment under microgravity (almost 4 days). Most of this 4-day period is used for a rather cumbersome transport of the Biobox facility by train from the laboratory in Moscow to the launch site in Plesetsk. During this period, Biobox was kept at 20 °C to minimize biological activity. Laboratory experiments (not published) showed that a 4-day culture period at ambient conditions is at the far end of what can be tolerated by the bone rudiments. These considerations indicate that the results of the Bion-10 experiment may be less reliable than the data of the IML-2 experiment. However, the Bion-10 data can be used, together with the data of a previous microgravity experiment on the IML-1 mission^11^, to evaluate the results of the IML-2 experiment. Combining the results of these three microgravity experiments with cultured fetal mouse long bones in evaluating the effect of microgravity on growth and matrix mineralization in skeletal tissues, as we are able to do here, was a unique opportunity in microgravity research.

This was the first study of the effects of spaceflight on ultrastructural aspects of fetal cartilage differentiation. Cell division was observed in both horizontal and vertical axes throughout the developing metatarsal, contrary to actual growth plates in which proliferation is more restricted to the vertical axis of the proliferative zone responsible for generating the cell columnar arrangement^26,27^. The more actively proliferating areas had less organized fibrils. The fibril orientation in all the zones of differentiation is not as defined as that observed in growth plates, probably due to the higher proliferation rate of embryonal stages, with the faster remodeling of extracellular matrix septa, which will not need/require long and thick fibrils. Such an effect has been documented in previous studies of the growth plate^26,27^. The most striking difference observed was between the baseline group, which represents the in vivo development, and the cultured groups. Prior to culture (Fig. 2B), the matrix is much better organized than after the rudiment is cultured (Fig. 2A). Proteoglycan granules (PGGs) differed slightly between the groups exposed to 1×*g*, either baseline (which appeared to have most), flight control or ground control, but in general, all of these groups had more granules than the microgravity group, indicating that there is an effect on PGGs due to microgravity exposure. A later in vivo study with mice exposed to 30 days of real microgravity in the Bion-M1 mission also reported a loss of proteoglycan staining in articular cartilage^28^.

The percentage increase in the overall length of the long bones (Fig. 3A; about 50%) is comparable with the growth found in the IML-1 experiment (55–60%)^11^. In both experiments no differences were found between the microgravity and flight control group and not between the flight control and the ground control groups. Also, in the Bion-10 experiment, no significant difference was found between the microgravity and the flight control group (Fig. 6A). The percentage increase in length in the Bion-10 experiment (11–17%) is much lower than in the IML-2 experiment (50%). It should be noted that the pre-flight measurement in Bion-10 was performed after the overnight pre-culture period, whereas this period was included in the IML-2 experiment. However, when the increase in overall length of the baseline group (15%) is subtracted from the IML-2 growth data in the Bion-10 experiment the growth rate is still half of that of the IML-2 experiment indicating sub-optimal culture conditions.

In this respect, it is not readily explicable that the growth found in the 6-h centrifuge group (IML-2) is reduced (Fig. 5A). However, Montufar-Solis and Duke^29^ reported from in vivo experiments that the length of the growth plate in rat tibia was reduced in response to hypogravity. In fact, they suggested a biphasic response with a decreased length of the growth plate under microgravity due to decreased differentiation and under hypergravity due to premature closure of the growth plate. It is possible that 1×*g* exposure for 6 h had comparable effects on our isolated fetal mouse long bones. Also, Stamenković and colleagues reported a reduced proliferation of cartilage cell structures after 16 days of microgravity^30^.

The results of the present study suggest that, because no changes were found in the length of the hypertrophic region (data not shown), microgravity per se does not interfere with normal cartilage proliferation and differentiation in isolated fetal mouse metatarsals. In ground-based experiments under intermittent hydrostatic loading, we found that the length of the hypertrophic region, measured in histological sections, was slightly, but significantly, increased in the loaded group^31^. It is interesting that a decreased length of the hypertrophic region is reported in the tibia of space-flown rats^26^. This discrepancy in the behavior of chondrocytes involved in the final differentiation process leading to the mineralization of the matrix should be studied in more detail in future microgravity experiments. In general, only limited data is available on the effects of microgravity on various cartilaginous tissues^32^, while this mechanically sensitive tissue deserves special attention regarding long durations of spaceflights.

Measurements of the increase in the length of mineralized diaphysis show that in the IML-2 experiment, microgravity significantly reduced the mineralization of the hypertrophic cartilage matrix. The results of the Bion-10 experiment also show a significant reduction in the mineralization under microgravity. These data are in accordance with an earlier microgravity experiment^11^ where we demonstrated in ED16 rudiments after 4 days of culture under microgravity a significant reduction in the length of the diaphysis (−31%) as well as in the uptake of ^45^Ca and ^32^P, -46% and −40%, respectively. The results of the three microgravity experiments indicate that cartilage involved in endochondral bone formation responds directly to microgravity and that microgravity reduces some aspects of cellular activity of hypertrophic chondrocytes resulting in decreased mineralization. This negative effect of microgravity on the mineralization process is also reported for the in vivo mineralization of bone^33,34^ during the same Bion-10/Cosmos 2229 mission where two rhesus monkeys (*Macaca mulatta*) were also exposed to 11 days of microgravity, and a reduction in mineralization parameters was reported in conjunction with increased bone resorption indicators^35^. Our data also complies with in vitro experiments in which microgravity is reported to decrease osteoblastic growth^10^ as well as a series of related growth factors^36^ and expression of the osteoblastic phenotype^8^. It is intriguing that in cultured fetal mice, long bones of the same age exposed to intermittent hydrostatic compression, mineralization of the hypertrophic cartilage is increased while the growth was not affected^31,37^. This could mean that there is a continuum in the response of chondrocytes to increasing strain levels with respect to mineralization. Although the underlying mechanism is still unclear, it is possible that changes in dilatational stress, probably resulting in changes in shear stress, especially in the transition zone between hypertrophic and mineralized cartilage^38,39^, are of crucial importance. However, it should be realized that, especially under in vitro conditions, the indirect effects of microgravity cannot be excluded completely^40^.

The suggestion from the present study, that the process of chondrocyte hypertrophy is not affected by microgravity because no changes were found in the length of the hypertrophic region is interesting, but should be studied in more detail in future experiments. For example, the proliferative potential of chondrocytes in an animal model for simulating microgravity, tail suspension, was decreased after 3 weeks of unloading^41^.

In this study, we used ED17 metatarsals, but we did not see any effects on osteoclasts/mineral resorption during the 4 days of culture. This contrasts with the increased mineral resorption we reported in our previous study^11^. However, in that previous study we used the very sensitive ^45^Ca tracing technique to identify osteoclastic activity while we also saw no obvious changes in osteoclasts cells. Direct effects of spaceflight on primary murine osteoclast were also reported by Tamma and colleagues^42^ via increased collagen type I fragments, increased levels of tartrate-resistant acid phosphatase (TRAP), and the increase of various bone resorption markers.

The varying periods of daily exposure to 1×*g* revealed that 6 h or more of 1×*g* exposure each day, fully counteracted the microgravity-related reduction in mineralization in this in vitro model. Multiple regression analysis showed that there is a significant relation between the mineralization and the time of 1×*g* exposure. This finding suggests a tissue memory for mechanical stimuli, which lasted for at least 18 h. In mechanically stressed avian long bones, changes in the orientation of matrix proteoglycans were reported, which persisted for more than 24 h in the absence of loading^15,16^. Also, bone marrow stromal cells from the tibia of rats, which were exposed to hind limb elevation for 5 days, showed a significantly reduced expression of osteocalcin mRNA after culture of up to 28 days^43^. This suggests that cultured cells and tissues retain a memory of their in vivo loading history which can be used in countermeasures to the unloading of microgravity. If indeed a cellular or tissue memory for the loading history exists, this may have implications for space-flight experiments where a 1×*g* onboard centrifuge is used. It may indicate that, at least in isolated skeletal tissues, stopping the centrifuge is necessary for manipulations of the experiment and may not seriously compromise the centrifuge samples as an on-board 1×*g* control. However, the parameters studied and the actual time course of expression of these parameters may also be of importance in this respect. In addition, the mechanically stressful launch should be taken into account. Cells and tissues might need an extended period of time to recover from launch effects before a sensible microgravity experiment can be performed^44^.

On the other hand, the fact that 3 h/day of 1×*g* exposure is not sufficient to counteract microgravity effects on mineralization and matrix composition, as shown in this in vitro study, is also worrying with respect to the efforts to apply short periods of artificial gravity, using a short arm human centrifuge (SAHC), as in-flight multi-system countermeasure in long duration missions^45,46^. With an Artificial Gravity (AG) exposure of 30 min of 2 g at foot level in a 2-meter radius system, one only applies 1.6% of the daily g-dose at heart level as countermeasure. This is even worse when we consider the exposure of the vestibular system, which in this case only receives about a 0.7% stimulus compared to regular physiological conditions on Earth^47^.

Currently, on the ISS crew members are scheduled for 2.5 h per day for countermeasure training^48^, and despite this significant duration, the efficacy of the treatment measured by aerobic and resistance exercise of 46 astronauts after long-duration mission indicated not to be fully protective against the near weightlessness multisystem deconditioning^49^. This lack of sufficient mechanical loading for the skeletal system also seems to have an impact on articular cartilage^50^.

As stated in the NASA Cross-Cutting Evidence Report^51^ under ‘Tests Needed to Close the Gaps’ it is stated that “It is not yet certain that exposure to centrifugation for intermittent, short periods of time is as beneficial as continuous exposure to normal gravity. Experiments must compare if it is the total block of time in AG or the number of AG exposures per day that is the most effective.” we may conclude from the current work that, at least for this in vitro developing bone system, we need more than 3 h a day of 1×*g* exposure in order to eliminate the microgravity effects.

Short-duration exposures in the order of half an hour per day seem not sufficient to reverse the effects of head-down tilt microgravity simulation for humans^47^. Such exposures cover only of few percent of the daily mechanical (gravity) load to the body. The more than 3 h of exposure time required for this in vitro study indicates that we might require longer duration or even chronic exposure to a 1×*g* stimulus for long-duration space flights as currently foreseen in order to provide the human body its required physiological mechanical environment^52^. A possible next step is to make use of translational research either on the ground to apply not a 24 h. a day reloading but only period-a-day exposure to 1×*g* for tail-suspended rodents (see e.g. ref. ^53^). Also, hypergravity experiment with, e.g. using zebra fish are promising models to decipher the role of (hyper-)gravity loading on articular cartilage^54^. For in-flight studies, a facility such as the Multiple Artificial-gravity Research System in the ISS Kibo module could very well be used for such studies^55^. Mice could be exposed for ‘short’ periods to 1×*g* per day to obtain the threshold for daily exposure for bone and cartilage, but also various other organ systems known to develop microgravity pathologies should be investigated. However, one might also more in detail revisit the concept(s) of in-flight chronic artificial gravity generated by rotating the complete spacecraft^56–58^.

## Methods

### Biorack facility

The experiment was performed in the European Space Agency (ESA) Biorack/Spacelab facility^59^ during the second International Microgravity Laboratory (IML-2) flown aboard the U.S. space shuttle Columbia (STS-65) launched 8 July 1994. As described earlier^11^, two identical Biorack models were used in this study: one was flown in Spacelab (Flight model) while the other model remained on the ground as a reference (Ground model). Biorack has a small radius (78 mm), slow rotating (107.5 ± 0.5 rpm) centrifuge providing a 1×*g*-reference condition while in microgravity (Flight control). The Flight and Ground experiments were each performed in standard Biorack Type-I/0 containers (20 × 40 × 80 mm).

### Tissue preparation and culture conditions

Isolated cartilaginous long bones (metatarsals, see also Fig. 4) from17 day embryonic Swiss random-bred mice (Harlan-Sprague-Dawley, Indianapolis, IN, USA) were cultured for 4 days using culture procedures and hardware as described earlier^11,23^ or Klein-Nulend et al. ^60^ and Houston et al. ^61^ for more details on this culture technique^60,61^. In brief, the metatarsals were cultured individually in gas-permeable, double-layered, polyethylene culture bags (2 × 4 cm), each containing 670 µl culture medium and a glass ampoule with 25 µl fixative (50% glutaraldehyde, final concentration 1.8%). Medium consisted of bicarbonate buffered alpha-modified essential medium (alpha-MEM) without nucleosides, supplemented with 50 mg/l of gentamicin, 0.5% v/v fungizone (Gibco, Rijswijk, The Netherlands), 0.2% BSA factor V, 50 mg/l l-ascorbic acid, 300 mg/l glutamine (Merck, Darmstadt, Germany) and 2 mM Na-ß-glycerophosphate (Sigma, St. Louis, MO, USA). After dissection, metatarsals were pre-cultured overnight in a 24-well plate (Greiner) in medium without Na-ß-glycerophosphate. The next day, metatarsals were transferred to culture bags, and 16 culture bags were placed in each Biorack container. Containers were then flushed with a gas mixture of 5% CO_2_/air and remained at room temperature for ~24 h during the handover of the containers to ESA and NASA officials, transportation to and storage in the shuttle, and subsequent launch. In the flown cultures, the µg-group and the 1×*g* flight control contained contralateral-paired bones. Also, the metatarsals of the 3-h and the 12-h groups were contralateral-paired.

Because of the long pre-launch incubation, an extra container kept at Kennedy Space Center (KSC) at the same pre-flight temperature conditions as the Flight and the Ground containers, was used as a baseline group to evaluate the conditions of the culture at the time of the initiation of the onboard experiment (baseline). When the Flight experiment began, the metatarsals of this baseline group were fixed and stored at 4 °C until further processing in the laboratory together with the other groups.

### Flight operations

After reaching orbit, the containers were flushed with a 5% CO_2_/air gas mixture and the experiment started by placing the Type-I containers in the 37 °C Biorack incubator. After 2 h, pressure build-up in the containers due to the temperature increase was released. The flushing procedure was repeated after 2 days.

To evaluate the effect of varying periods of daily 1×*g* exposure, three groups in addition to constant 1×*g* exposure were used: each was exposed daily to 1×*g* conditions by temporarily transferring the container from the µg-position to the 1×*g* on-board centrifuge for either 3, 6, or 12 h per day. The µg samples remained in the static positions in the incubator.

After 4 days, the astronauts ended the experiment by breaking the ampoules with fixative and then placing the containers in the +4 °C cooler. Post-landing, the containers were kept at +4 °C until further processing in the laboratory (see Table 1). Identical experiments were performed in the Ground model of Biorack 2 h after they were performed in space.

**Table 1:** IML-2—STS-65 time line

	Experiment activities and events
L−2 days	ED17 metatarsals were dissected; photomicrographs were taken; pre-culture at 37 °C (no Na-ß-glycerophosphate) o/n in standard 5% CO_2_ in an air incubator.
L−1 day	Medium change, metatarsals individually sealed into culture bags, Integration of the culture bags in the Type-I containers.
L−19 h	Handover of the experiment containers to ESA officials for inspection and later transport to the launch site in foam-isolated mid-deck locker inserts (ambient temperature).
L = 0 h	Launch of IML-2 (launched: KSC, Fl, USA, 8 July 1994).
L + 7 h	Activation of Biorack.
L + 13 h	Flushing of all containers with 5% CO_2_ in the air by the astronauts; start of the experiment.
L + 2 days 8 h	All containers were flushed again by the astronauts with 5% CO_2_ in the air.
L + 4 days 9 h	The astronauts terminated the experiment by breaking the glass ampoules with fixative and subsequently placing the containers in the +4 °C cooler.
L + 14 day 18 h	Landing of IML-2 at Kennedy Space Center; unloading of materials under temperature-controlled conditions.

### Biobox facility

A second experiment was performed on an unmanned Russian Biocosmos satellite, Cosmos-2229, launched on 29 December 1992/landed on 10 January 1993, in an incubator system called Biobox^62,63^. The microgravity portion of our experiment consisted of Embryonic Day 17 (ED17) metatarsals cultured in automated tissue culture modules made from polyethylene terephthalate, termed plunger boxes (PBs). The PBs (20 × 40 × 80 mm) use a system of plungers to move fluids (medium, fixative) from storage reservoirs to the culture compartment^64^ and were previously used for another microgravity experiment^24^. Eight plunger boxes were housed in closed cells in space (CIS) box^65^, which was integrated into Biobox and accommodated the microgravity samples.

For the in-flight 1×*g* control, 2 PBs were housed in Type-I/E containers, and placed on the Biobox centrifuge (radius 71.4 mm at the position of the samples). These Type-I/E containers were slightly modified to increase the internal volume used for the gas phase (5% CO_2_/air) to a volume/PB ratio comparable to the ratio in the CIS-container. After the integration of the PBs, the CIS box, as well as the modified Type-I/E containers, were flushed with 5% CO_2_/air. No additional flushing during the experiment occurred. After the CIS boxes were integrated into Biobox, and the Type-I/E containers placed on the centrifuge, Biobox was closed and kept at 20 ± 1.5 °C during transport, integration into the satellite, and launch (4 days in total). After reaching orbit, the temperature was automatically increased to 37 ± 1.5 °C, initiating the experiment (see Table 2), and the centrifuge started. The in-flight 1×*g* gravity field remained within 1.5% accuracy during the first 52 h of the flight. However, the speed of the centrifuge gradually increased up to a *g*-level of 1.4–1.5×*g* for the last 12 h of the experiment.

**Table 2:** BION-10—Cosmos 2229 timeline

	Experiment activities and events
L−5 days	ED17 metatarsals were dissected in laboratory in Moscow (Russia); pre-culture at 37 °C (no Na-ß-glycerophosphate) o/n in standard 5% CO_2_ in air incubator.
L−4 days	Photomicrographs are taken; loading the long bones in the hardware; hand over of exp. containers to ESA officials; integration of Biobox (temperature kept at 20 °C).
L−3 days	Transport (by train) of Biobox from Moscow to the launch site (Plesetsk, Russia) (Biobox temperature: 20 °C)
L−6 h	Integration of Biobox into the Biocosmos satellite.
L = 0 h	Launch of Bion-10 (Plesetsk, Russia).
L + 10 min	Start of warming up of Biobox to 37 °C.
L + 1 h	First automatic medium change.
L + 2 days	Second automatic medium change.
L + 4 days	The experiment was terminated by automatic fixation of the metatarsals; the temperature remained at 37 °C.
L + 9 days	Termination of all experiments in Biobox: lowering of the temperature to 14 °C.
L + 11 day 14 h	Landing of Bion-10 at Karaganda, Kazakhstan 10 January 1993; transport of Biobox to Moscow.
L + 14 day	Unloading of Biobox in Moscow; photomicrographs of the samples; preparations for shipment to home laboratory.

Identical experiments were performed in the Ground model of Biobox (however, lacking the 1×*g* centrifuge) two hours after they were performed in space.

### Tissue preparation and culture conditions

**Fig. 7:**
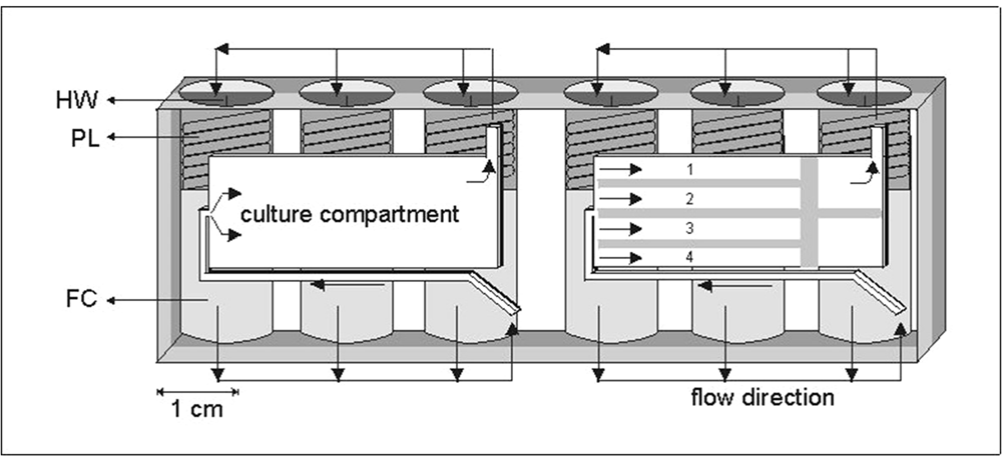
A schematic representation of an automated Plunger Box (PB) tissue culture module (20 × 40 × 80 mm (*l* × *d* × *h*)) made of a single block of polyethylene terephthalate (PET) as applied in the Bion-10/Cosmos-2229 mission. One module contains two culture compartments, 28 × 3 × 13 mm, each holding four metatarsal long bones. In a culture compartment, the long bones were separated from each other by small plates. For each culture compartment, fresh culture media or fixative were stored in three fluid compartments (FC). The fluid was forced to the culture compartment by moving a spring-loaded plunger (PL) released by scorching a nylon thread via a heated wire (HW). The fluid was led to the cultures via a system of internal channels and valves, indicated by arrows. The spent medium was forced out of the culture compartment and found its way to the now-void volume behind the just-released plunger. The culture compartments were covered by gas-permeable polyethylene foil restrained by a perforated metal plate. The Plunger Box system was developed by CCM, currently Sioux Technologies (Eindhoven, The Netherlands).

All pre-flight and some post-flight preparations were carried out in the ESA Moslab facility, at the Institute of Biomedical Problems (IBMP) in Moscow, Russia. 17-Day-old embryonic mouse metatarsal bones (Swiss random-bred mice, University of Leiden, The Netherlands) were pre-cultured overnight in a 24-well plate in medium (as above for IML-2) without Na-ß-glycerophosphate and then transferred to the plunger boxes (PBs). In each PB, there were two culture compartments, each covered with a gas-permeable membrane (polyethylene) and containing 1 ml of culture medium and four metatarsals. Storage reservoirs in the PBs contained medium and fixative (see Fig. 7). Medium was the same as in IML-2 (described above), except for 3 mM Na-ß-glycerophosphate (vs. 2 mM for IML-2). The medium was changed automatically upon reaching orbit and again after 2 days. At day 4 the metatarsals were automatically fixed with formaldehyde (final concentration 0.5%).

Post-flight, all metatarsals were removed from the PBs, washed in phosphate buffer, placed in 70% ethanol, and shipped at +4 °C from the laboratory in Moscow to our laboratory in Amsterdam, The Netherlands, for further processing experiment (see Table 2).

### Analysis

Photomicrographs of each individual metatarsal, taken immediately after dissection (IML-2) or just after the overnight culture (Bion-10), and again after return of the fixed samples, were used to measure overall length of the bones and length of the mineralized diaphysis as measure for newly deposited mineral. For histological studies, all samples were dehydrated and embedded in Historesin. Histological sections (3–4 µm) were stained with 0.2% toluidine blue (pH 5.2, no borax) (Fig. 1). For IML-2, some samples were also stained for tartrate-resistant acid phosphatase (TRAcP) to identify osteoclasts, and some were prepared for electron microscopy (Fig. 2). For ultrastructural analyses, the proximal cartilaginous ends, including a small band of the mineralized diaphysis, were post-fixed in 1% OsO_4_. Samples were decalcified in 10% EDTA (w/v) in 0.1 M Tris–HCl buffer, pH 7.4 at 4 °C, and embedded in Epon for sectioning. Thin sections were stained with uranyl acetate and lead citrate, and examined in a JEOL 100 CXII electron microscope. Micrographs were taken in the longitudinal septa of the reserve (RZ), proliferative (PZ), hypertrophy (HZ), and calcification (CZ) zones of 6–9 sections from each sample.

### Statistics

Statistical differences between the flight groups and the flight control and ground control were calculated using a two-tailed Student's *t*-test. For the varying 1×*g*-exposure periods, a polynomial regression analysis was performed. All data are expressed as mean ± SEM. The precise sample size is indicated in the legends of the figures, but was always equal to or >5.

## Data Availability

Some of the raw data is available on request with the corresponding author.
